# RNA-hydrolyzing activity of metallo-β-lactamase IMP-1

**DOI:** 10.1371/journal.pone.0241557

**Published:** 2020-10-30

**Authors:** Yoshiki Kato, Masayuki Takahashi, Mineaki Seki, Masayuki Nashimoto, Akiko Shimizu-Ibuka

**Affiliations:** 1 Department of Applied Life Sciences, Niigata University of Pharmacy and Applied Life Sciences, Niigata, Japan; 2 Research Institute for Healthy Living, Niigata University of Pharmacy and Applied Life Sciences, Niigata, Japan; Weizmann Institute of Science, ISRAEL

## Abstract

Metallo-β-lactamases (MBLs) hydrolyze a wide range of β-lactam antibiotics. While all MBLs share a common αβ/βα-fold, there are many other proteins with the same folding pattern that exhibit different enzymatic activities. These enzymes, together with MBLs, form the MBL superfamily. *Thermotoga maritima* tRNase Z, a tRNA 3′ processing endoribonuclease of MBL-superfamily, and IMP-1, a clinically isolated MBL, showed a striking similarity in tertiary structure, despite low sequence homology. IMP-1 hydrolyzed both total cellular RNA and synthetic small unstructured RNAs. IMP-1 also hydrolyzed pre-tRNA, but its cleavage site was different from those of *T*. *maritima* tRNase Z and human tRNase Z long form, indicating a key difference in substrate recognition. Single-turnover kinetic assays suggested that substrate-binding affinity of *T*. *maritima* tRNase Z is much higher than that of IMP-1.

## Introduction

Antibiotic-resistant bacteria, particularly Gram-negative strains, have developed a major defense mechanism against β-lactam-based antibiotics by producing β-lactamases (EC 3.5.2.6) that hydrolyze β-lactam rings. There are two groups of β-lactamases that exhibit different catalytic mechanisms: serine β-lactamases, which employ a serine as a catalytic residue, and metallo-β-lactamases (MBLs), which use one or two zinc ion(s) for catalysis. MBLs present a particular threat as they exhibit activity toward wide range of β-lactams including carbapenems, which are a class of β-lactams that are generally stable against serine β-lactamases. In addition, MBLs cannot be inhibited by mechanism-based inhibitors of serine β-lactamases [1, 2].

MBLs are further divided into three subclasses (B1, B2, and B3) based on their sequence similarities and structural features. More recent sequence- and structure-based phylogeny studies have indicated that subclass B1 + B2 and subclass B3 should be considered as separate MBL classes [3, 4]. With respect to substrate specificities, the B1 and B3 enzymes possess broad spectrums of activity, whereas the B2 enzymes act on a narrower substrate range. This difference in activity profiles may be associated with Zn^2+^ coordination differences at their catalytic sites as B1 and B3 enzymes exhibit maximum activity when two Zn^2+^ ions are bound to their active sites, but B2 enzymes are inhibited by the binding of more than one Zn^2+^ ion [1].

Subclass B1 includes the clinically important and transferable di-Zn MBLs. IMP-1, a subclass B1 enzyme, was first identified from *Serratia marcesens* in Japan in 1991, and since then, it has been found in several Gram-negative pathogenic bacteria [5, 6]. It was followed by the identification of variants, and now there are more than 50 IMP-type enzymes [7]. IMP-producing pathogens have spread throughout the world and threaten the clinical efficacy of β-lactam antibiotics [5, 8, 9]. The analysis of kinetic property revealed a broad substrate specificity of IMP-1, and its tertiary structure has been studied in detail [10, 11].

A wealth of structural data has revealed that many other proteins that share the characteristic MBL αβ/βα sandwich fold also exist, despite their highly diverged primary sequences. These are classed as members of the MBL superfamily. They include RNA processing proteins, DNA-uptake proteins, and hydrolases such as the MBLs and glyoxalase II [12, 13]. Having a conserved motif, they bind up to two metal ions in their active site, which are generally zinc, but some enzymes are reported to contain other ions such as iron and manganese ions [12, 14, 15]. tRNase Z (EC 3.1.26.11) is a MBL-superfamily enzyme that is expressed in almost all cells and catalyzes one of the reactions involved in tRNA maturation: the removal of a 3′-extension from pre-tRNA [16]. There are two forms of tRNase Z: a short form tRNase Z of 280–360 amino acids in length that is present in all three domains of life, and a long form that is found only in eukaryotes and is 750–930 amino acids in length [16]. Generally, a tRNase Z enzyme cleaves pre-tRNA immediately downstream of a discriminator nucleotide, which then becomes a substrate for tRNA nucleotidyltransferase-catalyzed CCA-addition reactions to generate mature tRNA. However, *Thermotoga maritima* tRNase Z (TmRZ) characteristically cleaves pre-tRNAs containing a _74_CCA_76_ sequence precisely after A_76_ to create mature 3′ CCA termini in a single step [16, 17]. Furthermore, TmRZ and some other tRNase Z enzymes can cleave unstructured RNAs (usRNAs) in addition to pre-tRNA. Two crystallographic studies have indicated that TmRZ binds one or two Zn^2+^ in its active site [18, 19]. However, catalytic analysis revealed that its activity was inhibited in the presence of Zn^2+^, while it was activated in the presence of Mn^2+^ or Mg^2+^ [20]. Hence the role of the metal ions in TmRZ is not totally understood.

Such a diversity of MBL enzymes, together with the fact that the MBL-superfamily enzymes exhibit a range of catalytic activities, prompted us to examine if MBLs could have other activities in addition to hydrolyzing β-lactam antibiotics. In this study, we analyzed IMP-1 hydrolytic activity toward RNA and DNA, and found that IMP-1 has an RNase activity. We discussed possible correlation between catalytic activities and structures of IMP-1 and TmRZ.

## Materials and methods

### Expression and purification of IMP-1

IMP-1 without its signal sequence and with a C terminal (His)_6_-tag was expressed in *E*. *coli* BL21(DE3)pLysS (Promega) harboring the plasmid pET28a-IMP-1Cth. This plasmid encodes the mature IMP-1 (UniProt ID: P52699) with a C-terminal (His)_6_-tag connected by a linker containing the thrombin recognition sequence. Transformed *E*. *coli* cells were grown in 100 mL aerated 2 × TY medium at 37°C to an OD_600_ of approximately 0.4–0.5, and the protein expression was induced with 0.1 mM isopropyl β-d-1-thiogalactopyranoside for 12 h at 18°C. Cells were then harvested by centrifugation, resuspended in 7 mL of 50 mM sodium phosphate buffer, pH 7.0, containing 500 mM NaCl, and disrupted by sonication. The supernatant obtained after centrifugation was loaded onto a 1 mL HisTrap FF column (GE Healthcare) pre-equilibrated with 50 mM sodium phosphate buffer containing 500 mM NaCl and then bound proteins were eluted with a 0–500 mM linear imidazole gradient. For salt removal and buffer exchange, the purified protein was loaded onto a PD-10 column (GE Healthcare) equilibrated with 20 mM HEPES buffer (pH 7.5) containing 50 μM ZnCl_2_ and eluted using the same buffer. To cleave the (His)_6_-tag, 3 U of thrombin (GE Healthcare) was added to every 1 mg of purified protein and the mixture was incubated at 10°C for 24 h. The thrombin was removed with a HiTrap Benzamidine FF column (GE Healthcare) equilibrated with 50 mM Tris-HCl buffer (pH 8.0) containing 500 mM NaCl. The eluate was then loaded onto a HisTrap FF column equilibrated with 50 mM sodium phosphate buffer (pH 7.0) and 500 mM NaCl to separate cleaved proteins from uncleaved (His)_6_-tagged proteins. The unbound protein was applied to a PD-10 column equilibrated with 20 mM HEPES buffer (pH 7.5) for buffer exchange and then concentrated with an Amicon Ultra 3K centrifugal filter to a final concentration of 5 mg/mL. The size and purity of the protein were confirmed by sodium dodecyl sulfate (SDS)-polyacrylamide gel electrophoresis (PAGE) on a 15% polyacrylamide gel. We also prepared a sample from the same *E*. *coli* strain harboring the pET-28a plasmid without any insert DNA by performing exactly the same steps as above, to rule out the possibility of contamination of any other nucleases.

### Preparation of tRNase Zs

The (His)_6_-tagged TmRZ and the (His)_6_-tagged human Δ30 tRNase Z long form (hTLΔ30) were prepared as described previously [17, 21]. The latter enzyme lacks the N-terminal 30 amino acids of the full-length tRNase Z, which are thought to correspond to the mitochondrial transport signal. TmRZ and hTLΔ30 used in this study were all (His)_6_-tagged.

### Total cellular RNA preparation and RNA-hydrolyzing assay

Total cellular RNA was prepared from the human multiple myeloma cell line KMM-1 using an RNAiso Plus kit (Takara Bio, Otsu, Japan). RNA (0.3 μg) was treated with 10 or 30 μg of IMP-1 in the presence of 10 mM MgCl_2_ or 10 mM MnCl_2_. After incubation at 37°C for 60 min, the solution was treated with an equal volume of phenol/chloroform to remove the enzyme, and the RNA was precipitated with ethanol and redissolved in distilled water. The RNA sample was then analyzed with an Agilent 2100 Bioanalyzer.

### *In vitro* usRNA and usDNA cleavage assay

RNA and DNA substrates were prepared as follows: a 24-nt RNA, usRNA1 (5′-GAGUGACUACCUCCAAGGCCCUUU-3′); a 22-nt RNA, usRNA9 (5′-GCCUGGCUGGCUCGGUGUAUUU-3′); and a 24-nt DNA, usDNA1 (5′-GAGTGACTACCTCCAAGGCCCTTT-3′) labeled with 5′-6-carboxyfluorescein were chemically synthesized and purified using high-performance liquid chromatography by JBioS (Saitama, Japan). Basically, 10 μg of enzyme was incubated with 10 pmol substrate and 10 mM MgCl_2_ at 37°C for 60 min. The enzyme concentration, the substrate concentration, the MgCl_2_ or MnCl_2_ concentration, the reaction time, and the reaction temperature were changed depending on the assay used. All experimental conditions are specified in the relevant figure legends. After the completion of the reactions, the mixtures were treated with an equal volume of phenol/chloroform to remove the enzymes, and the RNA or DNA was precipitated with ethanol, redissolved in distilled water, and mixed with an equal volume of sample buffer containing 10 mM Tris-HCl (pH 7.5), 1 mM EDTA, and 8 M urea. The reaction products were resolved on a 20% polyacrylamide–8 M urea gel. In reaction conditions where MgCl_2_ was replaced with MnCl_2_, electrophoresis was performed using a 20% polyacrylamide native gel. The gel was analyzed with a Gel Doc™ EZ Gel Documentation System (Bio-Rad).

### *In vitro* pre-tRNA 3′ processing assay

This assay was performed according to a previously described protocol [17]. Briefly, an 84-nt human pre-tRNA^Arg^ R-11TUUU (5′-GGGCCAGUGGCGCAAUGGAUAACGCGUCUGACUACGGAUCAGAAGAUUCCAGGUUCGACUCCUGGCUGGCUCG↓GUGUAAGCUUU-3′; the arrow denotes the cleavage site by hTLΔ30) was synthesized with T7 RNA polymerase (Takara Bio) from a corresponding synthetic DNA template and labeled with fluorescein at the 5′ end. This pre-tRNA sequence is originated from one of the human tRNA^Arg^ genes [22]. Next, 10 μg IMP-1 was incubated with 10 pmol substrate and 10 mM MgCl_2_. TmRZ and hTLΔ30 were used as positive controls. All reactions were incubated at 37°C for 60 min for IMP-1 and TmRZ and for 30 min for hTLΔ30. Following the completion of the reactions, the mixtures were treated as described above and analyzed by electrophoresis on an 8 M urea/20% polyacrylamide large gel.

### Kinetic analysis

To determine *k*_chem_ (rate constant) and *K*_d_ (dissociation constant) for the RNase activity of IMP-1 and TmRZ, single-turnover assays were performed, in which *k*_obs_ (apparent rate constant) was measured at different enzyme concentrations [23]. usRNA9 and usRNA1 were used to determine the constants for IMP-1 and TmRZ, respectively. IMP-1 at a final concentration of 4, 10, or 20 μM was incubated with 0.5 μM usRNA9 in the presence of 10 mM MgCl_2_ at 37°C for 10–30 min. TmRZ in a final concentration of 0.1, 0.15, 0.2, or 0.4 μM was incubated with 0.05 μM usRNA1 in the presence of 10 mM MnCl_2_ at 37°C for 3–9 min. The band of the single cleaved product was quantified by the analysis tool of Gel Doc^TM^ EZ Gel Documentation System, to calculate the concentration of the product in each condition. The *k*_obs_ was calculated from the following equation:

$$P=S_{0}(1-e^{-k_{obs}∙t}),$$

where *P* is the concentration of the cleaved product at time *t*, *S*_0_ is the initial concentration of the used substrate, and *t* is time (min). The *k*_obs_ value was plotted against the enzyme concentration, and the graph was fitted with the following equation using Kaleida Graph (Synergy Software) to obtain *k*_chem_ and *K*_d_:

$$k_{obs}=k_{chem}∙E_{0}/(E_{0}+K_{d}),$$

where *E*_0_ is an enzyme concentration (μM).

### Structural comparison between IMP-1 and TmRZ

The crystal structures of IMP-1 and TmRZ were compared using the Dali web server pairwise comparison tool (http://ekhidna2.biocenter.helsinki.fi/dali/) [24]. The IMP-1 amino acid residues were designated according to the standard numbering scheme for MBLs [25, 26].

## Results and discussion

### Hydrolytic activity of IMP-1 against total human cell RNA and unstructured RNAs

Structural similarity between IMP-1 and TmRZ led us to analyze the RNase activity of IMP-1. First, we examined the hydrolysis activity of purified IMP-1 against total human cell RNA (S1 Fig in S1 File). IMP-1 was observed to hydrolyze both 18S and 28S rRNAs into smaller RNAs.

**Fig 1:**
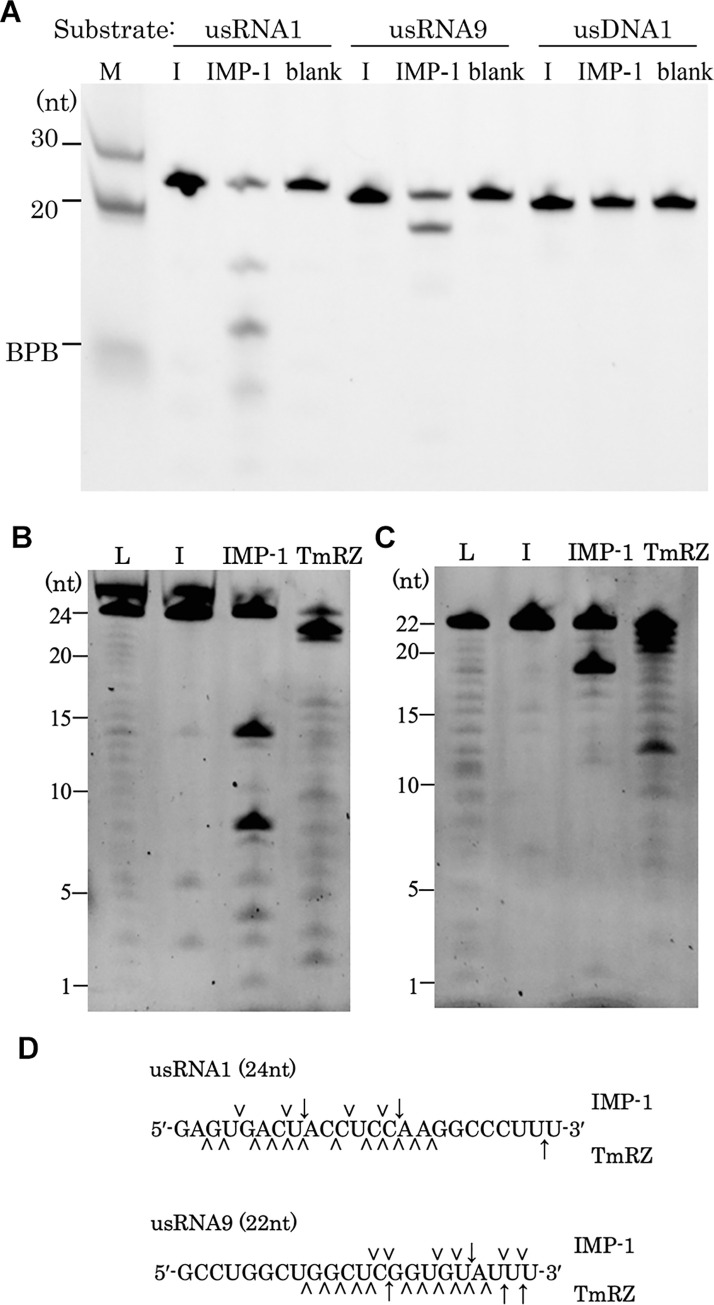
Cleavage assay of unstructured RNA and DNA substrates. (A) Cleavage of usRNAs in the absence (-) and presence of IMP-1. Each substrate (10 pmol) was incubated with 10 μg IMP-1 and 10 mM MgCl_2_ at 37°C for 60 min and then analyzed on a denaturing gel. Lane M: 30- and 20-nt markers and bromophenol blue (BPB) that migrates at an approximate 8-nt position. In the reaction for the lane “blank”, the enzyme was replaced with the sample prepared from the *E*. *coli* cells harboring the empty plasmid pET-28a. Determination of cleavage sites in (B) usRNA1 and (C) usRNA9. In the TmRZ reaction, MnCl_2_ (10 mM) was added instead of MgCl_2_ (10 mM). The IMP-1 reaction was performed for 60 min at 37°C, and the TmRZ reaction was carried out for 30 min at 50°C. (B) usRNA1 cleavage products and (C) usRNA9 cleavage products are shown on denaturing gels. Lane L: ladder marker; lane I: input substrate RNA. (D) Summary of the cleavage sites estimated from the cleavage product electrophoretic migration patterns. The IMP-1 and TmRZ cleavage sites are shown above and below the nucleotide sequences, respectively. Major cleavage sites are shown by arrows, and minor sites are indicated by arrowheads.

TmRZ and some other tRNase Zs are known to cleave small unstructured RNAs (usRNAs) in addition to pre-tRNA [27]. Therefore, we investigated the hydrolytic activity of IMP-1 against two 5′-6-carboxyfluorescein-labeled usRNAs: 24-nt usRNA1 and 22-nt usRNA9. IMP-1 was able to cleave both usRNAs, but it did not show any hydrolyzing activity toward usDNA1 (Fig 1A). We performed further usRNA cleavage assays to specify which sites were cleaved by TmRZ and IMP-1 (Fig 1B and 1C). Analysis of the digestion patterns revealed that IMP-1 tended to cleave substrates after U or C nucleotides and preferentially between U/C and A nucleotides (Fig 1D).

**Fig 2:**
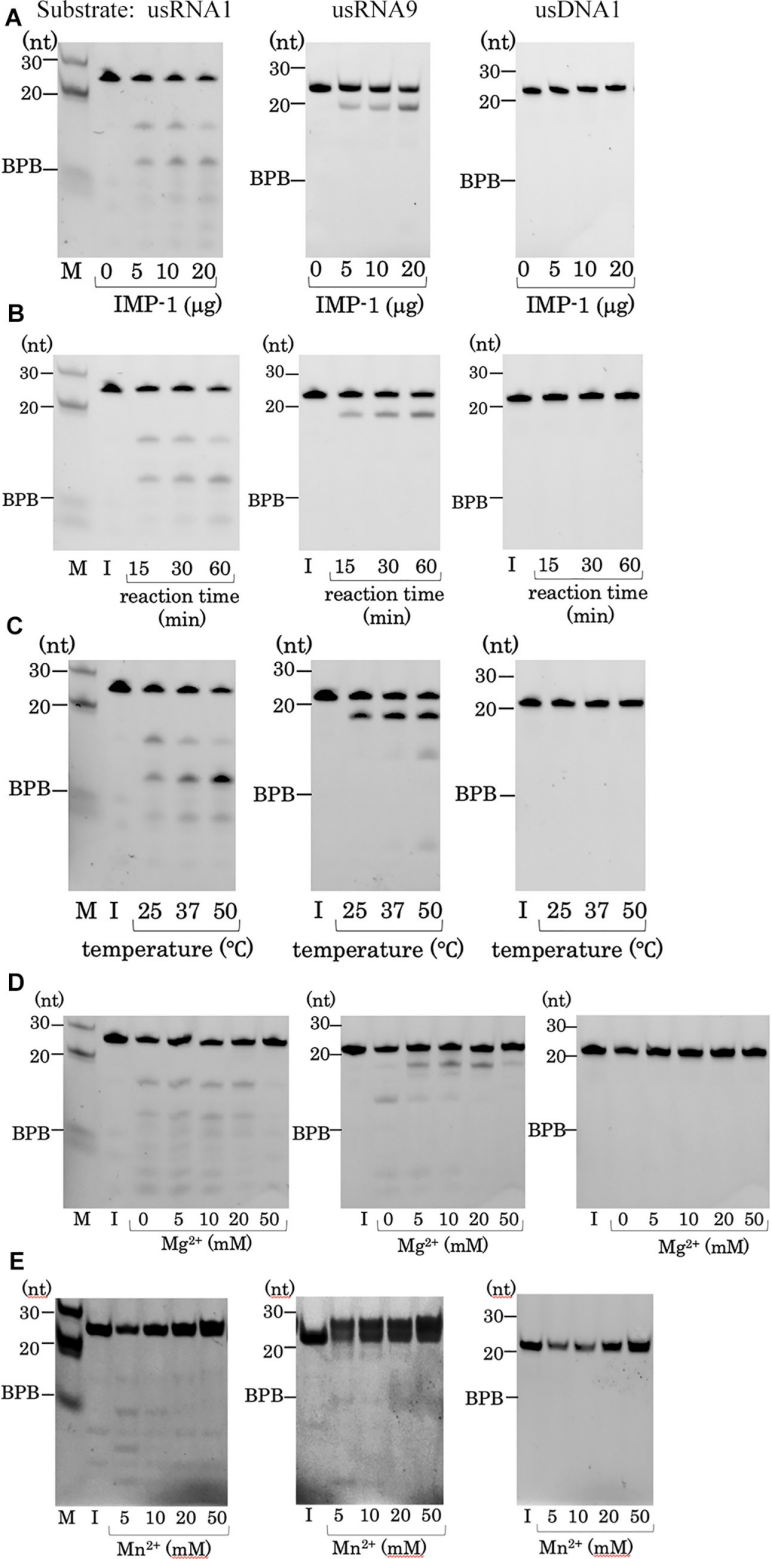
IMP-1 RNA cleavage activities under various reaction conditions. In each panel, three gel images are shown for usRNA1, usRNA9, and usDNA1 (from left to right). The assays were performed to investigate the effects of (A) enzyme concentration, (B) reaction time, (C) reaction temperature, (D) Mg^2+^ concentration, and (E) Mn^2+^ concentration. Lane M: 30- and 20-nt markers and BPB that migrates at an approximate 8-nt position. Lane I: input substrate RNA.

We investigated the optimal conditions for IMP-1-mediated usRNA cleavage by performing assays using varied enzyme concentrations, reaction times, reaction temperatures, and Mg^2+^ or Mn^2+^ concentrations (Fig 2). Although RNA hydrolysis was observed under most of the conditions tested, DNA hydrolysis was not detected at all, indicating that IMP-1 does not have any DNase activity. The final yields of cleaved usRNAs were dependent upon the enzyme concentration used (Fig 2A). The total yields of cleavage products were observed to increase in a time-dependent manner (Fig 2B), and they also increased as the reaction temperature was increased up to 50°C (Fig 2C). The RNA substrates were barely hydrolyzed at 80°C, and no products were detected for usDNA1 (data not shown). This observation is consistent with the known thermal stability of IMP-1. The IMP-1 β-lactamase activity was maintained even at 60°C, but it decreased gradually at temperatures above 60°C and was lost completely by a 30 min incubation of the enzyme at 80°C (unpublished data).

The effect of Mg^2+^ was substrate-dependent (Fig 2D). IMP-1 was able to hydrolyze usRNA1 even in the absence of Mg^2+^, and the quantities and sizes of the generated fragments were similar throughout a range of MgCl_2_ concentrations (0–20 mM). However, the usRNA9 cleavage pattern was different in the absence of Mg^2+^ compared with when Mg^2+^ was present. For the both substrates, IMP-1 activity was inhibited in the presence of 50 mM Mg^2+^.

The RNase activity of IMP-1 was also examined in the presence of a range of Mn^2+^ concentrations. For these particular assays, we omitted the phenol/chloroform treatment step to remove enzymes from the reaction mixtures as the RNAs were not well recovered by this treatment. It suggests that strong complexes are formed between the enzyme and its substrates in the presence of Mn^2+^. This presumption is supported by the fact that addition of Mn^2+^ to a reaction mixture caused the input RNA to migrate more slowly on a gel, which was predominantly observed when usRNA9 was used as the substrate (Fig 2E). usRNA1 was cleaved in the presence of 5 and 10 mM Mn^2+^, and usRNA9 was cleaved in the presence of 5 mM Mn^2+^, but the IMP-1 activity was inhibited in the presence of higher concentration of Mn^2+^. Mg^2+^ and Mn^2+^ ions have been reported to activate TmRZ over a concentration range of 0.02–10 mM [20]. This report suggested that Mg^2+^ or Mn^2+^ could be coordinated near the catalytic center to induce an active-enzyme/substrate conformation, and that the Zn^2+^ ions in the catalytic center of TmRZ could be replaced with Mg^2+^ or Mn^2+^ upon substrate binding to establish such a conformation. The RNase activity of IMP-1 was not detected in the presence of 50 μM Zn^2+^, which is the concentration used for the analysis of β-lactamase activity, indicating the difference in metal ion requirement between of RNA and β-lactam hydrolysis. In this condition, no RNA was recovered by the phenol/chloroform extraction, as in the condition with Mn^2+^ (data not shown). In a common reaction scheme for subclass B1 MBL, it is suggested that two zinc ions are mandatory for the hydrolysis of β-lactams, while the scheme for tRNase Z and the role of the metal ions are not completely understood (S2 Fig in S1 File) [2, 20, 28]. As for TmRZ, their roles may be such that endogenous Zn^2+^ ion(s) is essential for catalysis and that supplemental Mg^2+^ ions strengthen the enzyme/substrate interaction. Though the effects of these metal ions were different between TmRZ and IMP-1, our results show that the metal ions do affect the RNase activity of IMP-1 by changing the binding state between the enzyme and the substrates. The β-lactamase activity of IMP-1 was not largely affected by addition of 50 μM Zn^2+^, Mn^2+,^ or Mg^2+^, and slightly increased by the addition of higher concentration (10 mM) of any metal ion (data not shown).

### Hydrolytic activity of IMP-1 against pre-tRNA^Arg^

**Fig 3:**
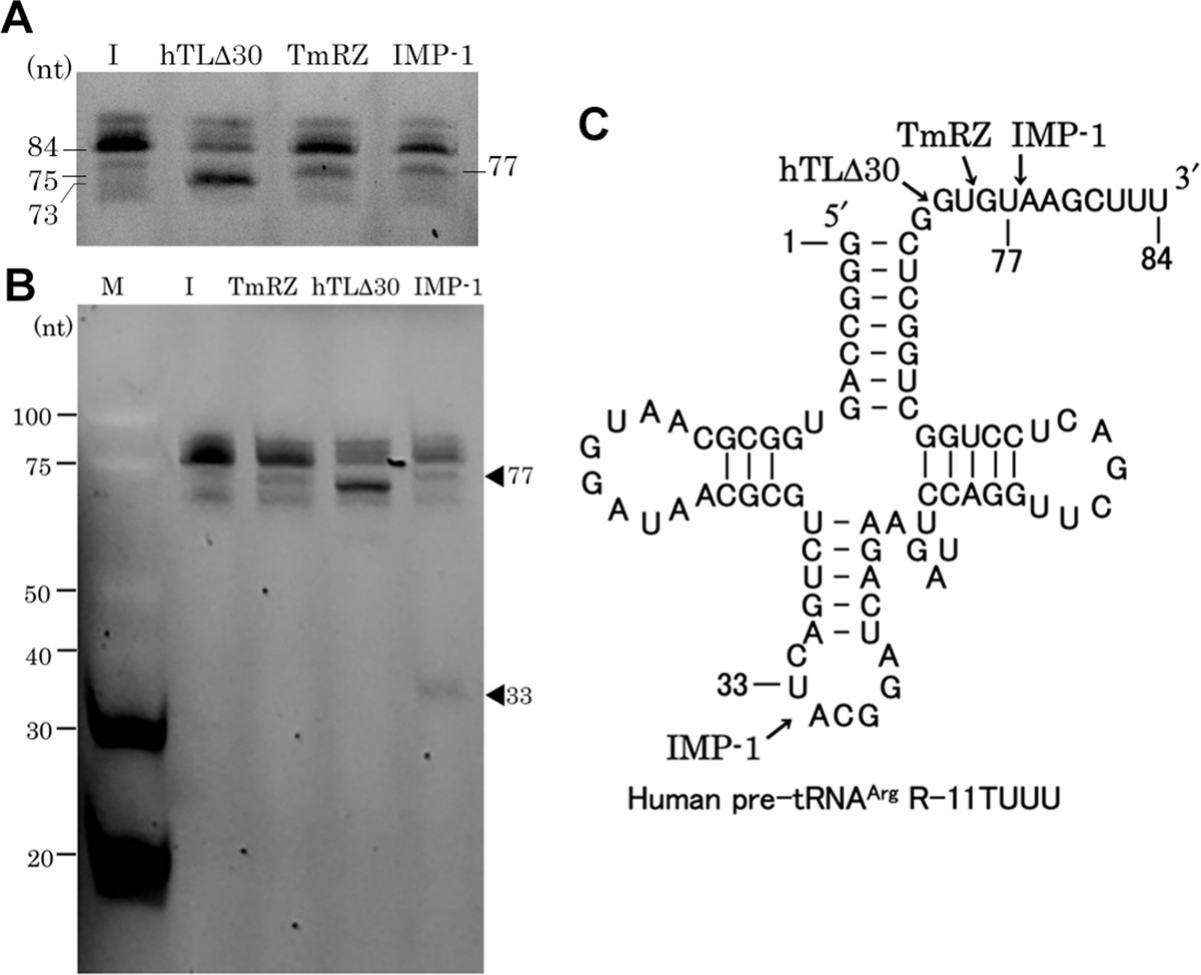
Cleavage of human pre-tRNA^Arg^ by IMP-1, TmRZ, and hTLΔ30. A partly enlarged gel image (A) and the whole gel image (B) are shown. All reactions were performed at 37°C for 60 min for TmRZ and IMP-1 and 30 min for hTLΔ30. Lane I: input substrate R-11TUUU; lane M: size markers. In panel B, fragments produced by IMP-1 cleavage are indicated with arrowheads. (C) Secondary structure of human pre-tRNA^Arg^ R-11TUUU. Arrows indicate the enzyme cleavage sites.

We assessed the hydrolytic activity of IMP-1 against pre-tRNA^Arg^ (Fig 3). hTLΔ30 and TmRZ were used as positive controls as hTLΔ30 is known to cleave this pre-tRNA after the G at nt 73 and TmRZ cleaves it after the U at nt 75 [17, 22]. IMP-1 was observed to cleave pre-tRNA^Arg^ into two fragments, and the larger product was generated by its cleavage after the U at nt 77 (Fig 3). The smaller fragment migrated on a gel between the 30-nt and 40-nt markers, suggesting that a cleavage occurred in the UA sequence in the anticodon-loop to generate a 33-nt fragment (Fig 3B and 3C). This is consistent with our usRNA cleavage assay data that indicated that IMP-1 cleaves substrate RNAs preferentially between U/C and A (Fig 2).

### Kinetic analysis of the IMP-1 RNase activity

Steady state kinetic analysis of the IMP-1 RNase activity needs to quantitate the substrate/product at low enzyme concentrations, which was difficult here due to the detection limit of the assay method. As an alternative, we performed single-turnover assays and determined *k*_chem_ and *K*_d_ values (Table 1). Though the error of the IMP-1 *k*_chem_ was large, the *k*_chem_ values of IMP-1 and TmRZ were within the same order of magnitude, showing that these enzymes catalyze the cleavage of the substrate molecules with comparable rate. On the other hand, the *K*_d_ value of IMP-1 was > 200-fold higher than that of TmRZ, indicating that the affinity of IMP-1 toward the substrate was much lower than that of TmRZ. However, we should note that the substrate of IMP-1 was usRNA9 and that the substrate of TmRZ was usRNA1, for the reason that each enzyme generated a single major cleavage product for the selected substrate. This difference of substrate could be a cause of the observed difference in *K*_d_ values.

**Table 1:** Kinetic parameters obtained from single-turnover assay.

Enzyme (Substrate)	IMP-1 (usRNA9)	TmRZ (usRNA1)
*k*_chem_ (sec^-1^)	0.015 ± 0.0005	0.0038 ± 0.0015
*K*_d_ (M)	5.9×10^−5^ ± 2.7×10^−5^	2.1×10^−7^ ± 1.7×10^−7^
*k*_chem_/*K*_d_ (sec^-1^M^-1^)	2.5×10^2^ ± 1.5×10^2^	1.8×10^4^ ± 1.6×10^4^

### Structural comparison of IMP-1 and TmRZ

**Fig 4:**
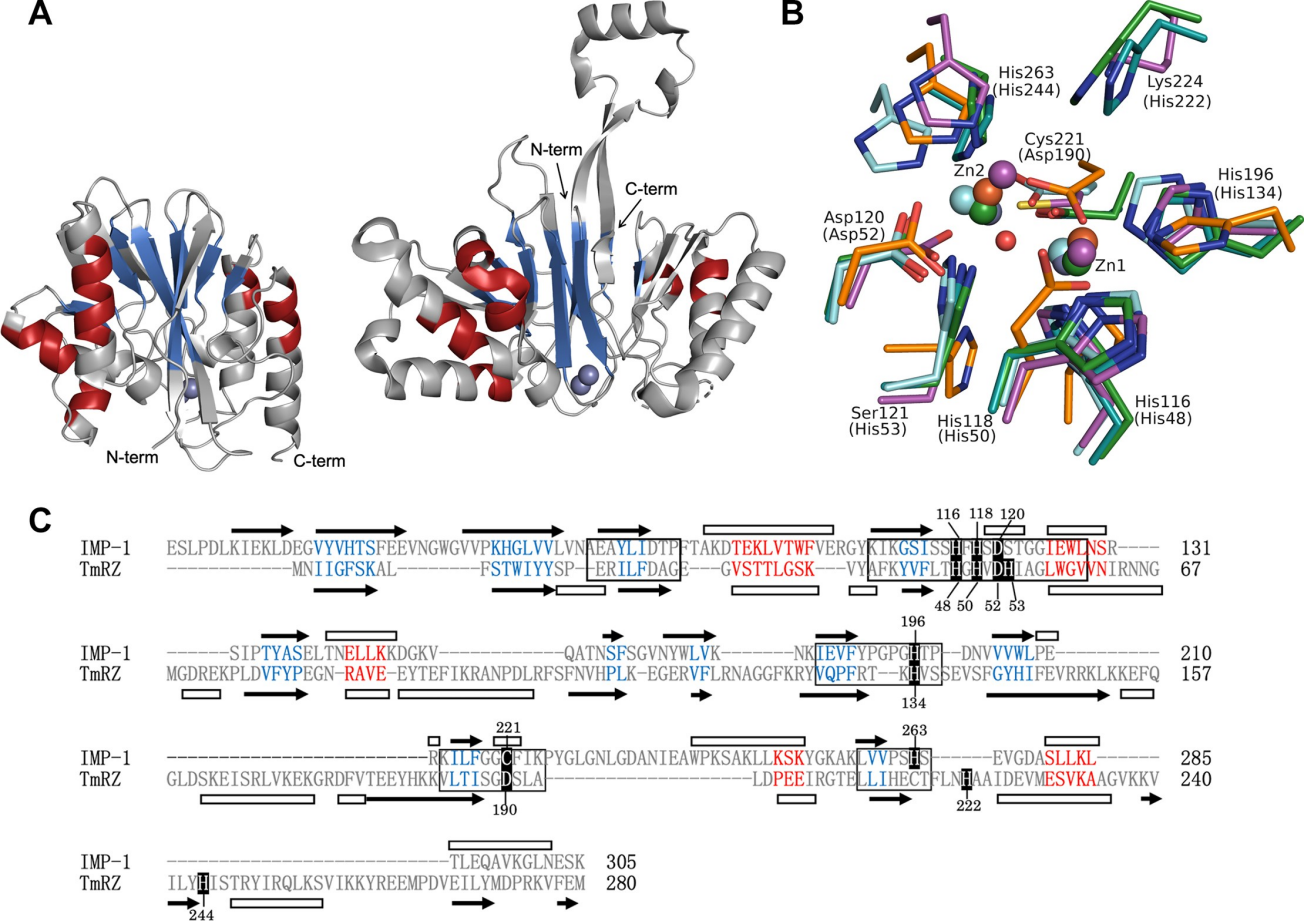
Comparison of the tertiary structures of IMP-1 and TmRZ, and structure-based alignment. (A) Overall structures of IMP-1 (left) and TmRZ (right) in the same orientation. (B) Comparison of metal-binding active sites of MBL superfamily enzymes: IMP-1 with Zn^2+^ (violet), TmRZ with Zn^2+^ (blue), quinolone signal response protein PqsE with Fe^3+^ (2Q0I, cyan), rubredoxin oxygen:oxidoreductase with Fe_2_O (1E5D, orange), and FAD photoproduct diphosphatase with Mn^2+^ (6B9V, green). Metal ions and an oxygen of Fe_2_O are shown as spheres. The amino acids involved in the coordination of Zn^2+^ are labeled with and without parentheses to indicate residues from TmRZ and IMP-1, respectively. (C) Alignment of primary structures based on a structural comparison using the Dali algorithm [24]. Secondary structures are indicated by black arrows (β-strands) and white bars (α-helices). The five conserved segments of the MBL superfamily are boxed. Residues coordinated to zinc ions are shown in white letters against a black background, with their residue numbers. In the figures A and C, the corresponding secondary structure elements are colored in red (α-helix) and in blue (β-strand). The figures A and B were generated by The PyMOL Molecular Graphics System, Version 2.0 (Schrödinger, LLC).

To further investigate differences between IMP-1 and TmRZ, we compared their structures. The coordinate files for the crystal structures of IMP-1 (PDB ID:1DDK) [11] and TmRZ (2E7Y, chain A) [19] contained data for 220 and 269 residues, respectively, and the RMSD value between these two structures was 3.3 Å for the Cα atoms of the 141 residues structurally aligned by Dali program. The core αβ/βα domains of these enzymes can be superimposed, though there are some mismatched secondary structure elements (Fig 4A). TmRZ also possesses a protruding flexible arm, which does not exist in IMP-1. Comparison of catalytic sites of MBL superfamily enzymes indicates that the positions of metal ions are analogous, though the position of metal ions corresponding to Zn2 of IMP-1 is a little diverse (Fig 4B). The positions and the orientations of the metal-coordinating residues are also reasonably conserved, with some exceptions. In IMP-1 and TmRZ, most of the Zn^2+^-coordinating residues were in MBL superfamily conserved regions, but His222 and His244 of TmRZ are located outside of these regions (Fig 4C).

### Evolutionary aspects of the dual function of IMP-1

IMP-1 possessed a reasonably strong hydrolytic activity toward RNA substrates, revealing that it has an additional catalytic function that had not been previously defined. Baier *et al*. reported that several MBL-superfamily enzymes can possess multiple catalytic activities. These include several non-native MBL reactions that have never been catalyzed by MBL-superfamily enzymes [29]. PNGM-1, which was isolated from a functional metagenomic library from deep-sea sediments from Edison Seamount, was recently reported to exhibit both β-lactamase and endoribonuclease activities, even though the *k*_cat_ values of its β-lactamase activity were 10^3^–10^4^-fold lower than those of usual clinically identified MBLs [30]. It seems plausible that a basic catalytic mechanism is conserved in the MBL-superfamily enzymes and that each enzyme is customized for a certain substrate by a particular arrangement of the residues surrounding the active site, the addition of extra loops or domains, and through a specific metal ion-binding mode. We measured the β-lactamase activity of TmRZ with several substrates in the presence of Zn^2+^, Mg^2+^, or Mn^2+^, but no hydrolysis was detected (data not shown). This implies narrower substrate specificity of TmRZ, and/or difference in the metal requirement between MBL and TmRZ.

Our results suggest that IMP-1 produced by antibiotic-resistant bacteria will not only hydrolyze β-lactams but may also cause unexpected pathological effects through its RNase activity in patients suffering from a bacterial infection. We are currently investigating how the RNase activity of IMP-1 affects bacterial cells and human cells physiologically and pathologically. In addition, we are planning to conduct detailed investigations of the catalytic and structural properties of more MBL-superfamily members with a view to their future utilization as *de novo* catalysts.

## Supporting information

S1 File (DOCX)
