# Supplementary material for: RNA-hydrolyzing activity of metallo-β-lactamase IMP-1
Source: PLoS One. 2020 Oct 30;15(10):e0241557. doi: 10.1371/journal.pone.0241557 (PMC7599082; doi:10.1371/journal.pone.0241557)
Supplement: S1 File — (DOCX) [file pone.0241557.s001.docx]

**Supplementary data**

**A**


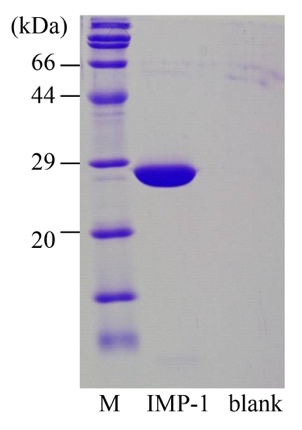


**B C**


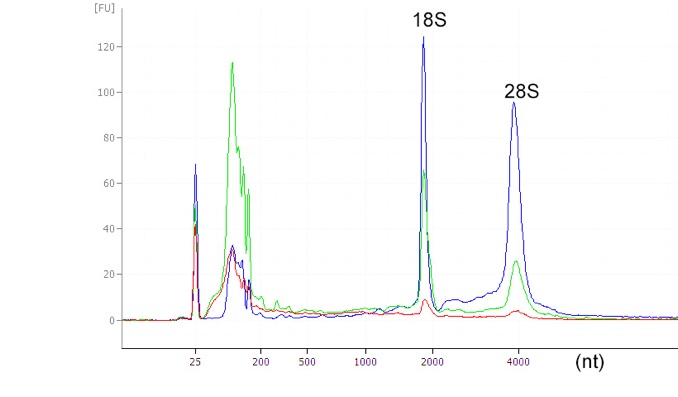

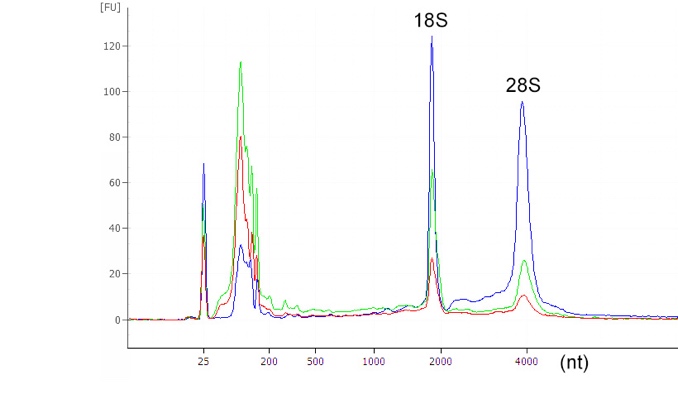


**Supplementary fig 1.** IMP-1 RNA-hydrolyzing activity on total human cell RNA. (A) SDS-PAGE of the purified IMP-1 that was used for the analyses. Lane M shows molecular weight markers, and 5 µg of the purified enzyme is loaded in lane IMP-1. Lane “blank” is the sample prepared from the *E. coli* cells harboring the empty plasmid pET-28a, as described in the Materials and Methods section. Panels B and C show the IMP-1-catalyzed hydrolysis of total human cell RNA that was measured using the Agilent 2100 Bioanalyzer. In both panels, the vertical axis indicates signal intensity (fluorescence units) and the horizontal axis indicates fragment size. The blue lines indicate reaction mixtures without the addition of IMP-1. (B) Reactions with 10 µg IMP-1 (green) or 30 µg IMP-1 (red) in the presence of 10 mM MgCl_2_. (C) Reactions with 10 µg IMP-1 in the presence of 10 mM MgCl_2_ (green) or 10 mM MnCl_2_ (red).

**A B**


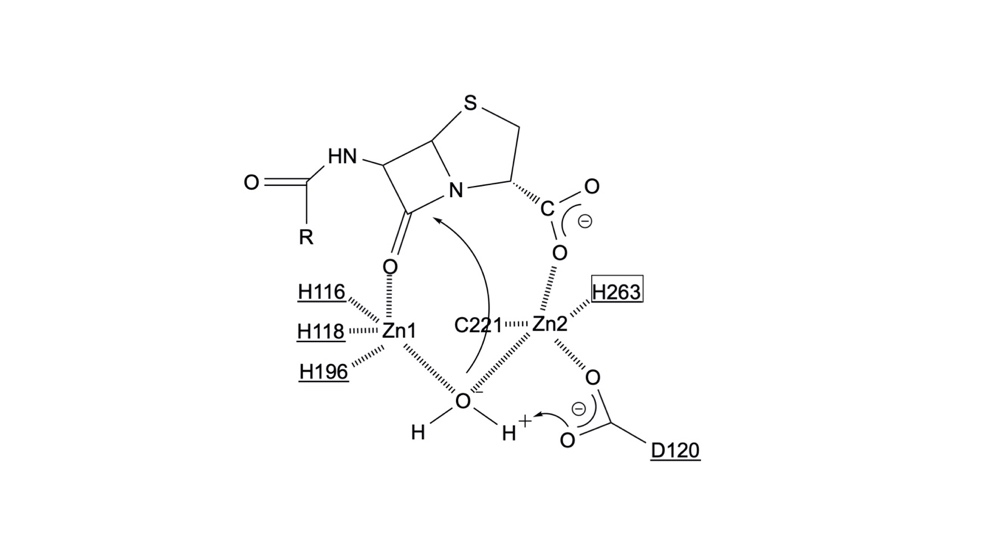

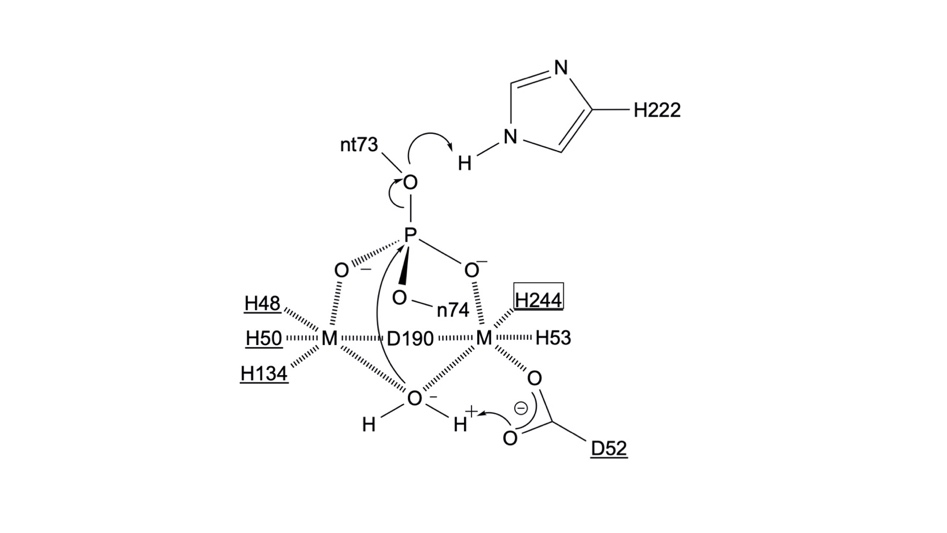


**Supplementary fig 2.** Schemes for the reaction mechanism of MBL and tRNase Z. Schemes for the catalytic mechanisms of (A) subclass B1 MBL and (B) tRNase Z, based on the published works [1-3]. Residue numbers are those of IMP-1 and TmRZ, and the conserved residues and structurally corresponding residues between IMP-1 and TmRZ are underlined and boxed, respectively, in each panel. “M” in the panel B indicates a metal ion possibly bound to the active site of tRNase Z.

1. Palzkill T. Metallo-β-lactamase structure and function. Ann N Y Acad Sci. 2013;1277:91-104. doi: https://doi.org/10.1111/j.1749-6632.2012.06796.x PMID: 23163348.

2. Minagawa A, Takaku H, Ishii R, Takagi M, Yokoyama S, Nashimoto M. Identification by Mn^2+^ rescue of two residues essential for the proton transfer of tRNase Z catalysis. Nucleic Acids Res. 2006;34(13):3811-8. doi: https://doi.org/10.1093/nar/gkl517 PMID: 16916792.

3. Li de la Sierra-Gallay I, Pellegrini O, Condon C. Structural basis for substrate binding, cleavage and allostery in the tRNA maturase RNase Z. Nature. 2005;433(7026):657-61. doi: https://doi.org/10.1038/nature03284 PMID: 15654328.
